# Evolutionary and genomic analysis of four SARS-CoV-2 isolates circulating in March 2020 in Sri Lanka; Additional evidence on multiple introduction and further transmission

**DOI:** 10.1017/S0950268821000583

**Published:** 2021-03-16

**Authors:** Dilan Amila Satharasinghe, Parakatawella Mudiyanselage Shalini Daupadi Kumari Parakatawella, Jayasekara Mudiyanselage Krishanthi Jayarukshi Kumari Premarathne, L. J. P. Anura P. Jayasooriya, Gamika A. Prathapasinghe, Swee Keong Yeap

**Affiliations:** 1 Department of Basic Veterinary Science, Faculty of Veterinary Medicine and Animal Science, University of Peradeniya, Peradeniya 20400, Sri Lanka; 2 Department of Livestock and Avian Science, Faculty of Livestock, Fisheries and Nutrition, Wayamba University of Sri Lanka, Makandura 60170, Sri Lanka; 3 China ASEAN College of Marine Sciences, Xiamen University Malaysia, Sepang 43900, Malaysia

**Keywords:** COVID-19, molecular evolution, phylogeny, SARS-CoV-2, Sri Lanka

## Abstract

The molecular epidemiology of the virus and mapping helps understand the epidemics' evolution and apply quick control measures. This study provides genomic evidence of multiple severe acute respiratory syndrome coronavirus 2 (SARS-CoV-2) introductions into Sri Lanka and virus evolution during circulation. Whole-genome sequences of four SARS-CoV-2 strains obtained from coronavirus disease 2019 (COVID-19) positive patients reported in Sri Lanka during March 2020 were compared with sequences from Europe, Asia, Africa, Australia and North America. The phylogenetic analysis revealed that the sequence of the sample of the first local patient collected on 10 March, who contacted tourists from Italy, was clustered with SARS-CoV-2 strains collected from Italy, Germany, France and Mexico. Subsequently, the sequence of the isolate obtained on 19 March also clustered in the same group with the samples collected in March and April from Belgium, France, India and South Africa. The other two strains of SARS-CoV-2 were segregated from the main cluster, and the sample collected from 16 March clustered with England and the sample collected on 30 March showed the highest genetic divergence to the isolate of Wuhan, China. Here we report the first molecular epidemiological study conducted on circulating SARS-CoV-2 in Sri Lanka. The finding provides the robustness of molecular epidemiological tools and their application in tracing possible exposure in disease transmission during the pandemic.

## Introduction

The severe acute respiratory syndrome coronavirus 2 (SARS-CoV-2) emerged in the late 2019s, causing coronavirus disease 2019 (COVID-19). The SARS-CoV-2 virus spread rapidly throughout the world with an unsettling effect on human livelihood and economy. Globally, since 31 December 2019 and as of week 2021-5, 106 472 660 cases of COVID-19 have been reported, including 2 323 103 deaths. Since 31 December 2019 and as of the fifth week, 2021, >106 472 660 confirmed cases, including 2 323 103 deaths, have been reported worldwide, affecting >343 countries as indicated by the European Centre for Disease Prevention and Control [1]. The World Health Organization (WHO) has declared a global health emergency at the end of January 2020 [2]. In Sri Lanka, the first local case of COVID-19 was recorded on 11 March 2020 in a 58-year-old male. As of 23 February 2021, there were 80 517 confirmed cases of COVID-19, including 450 deaths reported to the WHO [3].

A robust surveillance system, understanding the molecular epidemiology of the virus, and mapping can help to understand the evolution of the epidemics and apply quick control measures [4, 5]. Currently, based on the genomic epidemiology mapping of the COVID-19 virus, it has been demonstrated that the virus is undergoing mutations [6]. Therefore, in addition to confirmation of the presence of the virus, the WHO recommends regular sequencing of a percentage of specimens from clinical cases to monitor viral genome mutations that might affect the medical countermeasures, including diagnostic tests [5]. The whole genome of four SARS-CoV-2 virus strains obtained from COVID-19 positive local patients was sequenced and deposited in the Global Initiative on Sharing All Influenza Data (GISAID) EpiCoV™ database. This study was conducted to investigate the evolution and genetic relatedness of SARS-CoV-2 strains in Sri Lanka, with other reported SARS-CoV-2 strains.

## Materials and methods

### Sri Lankan SARS-CoV-2 sequences

The whole-genome sequence of four SARS-CoV-2 virus strains obtained from COVID-19 positive patients, including the first local positive case reported on 11 March 2020, was deposited in the GISAID EpiCoV™ database^5^ were used for this study (Table 1).

**Table 1.** Four Sri Lankan SARS-Cov-2 virus strains available on the GISAID EpiCoV™ database

Virus name	Passage history	Patient details/history	Accession ID	Collection date	Length	Host	Specimen source	Originating lab	Acknowledgement
hCoV-19/Sri Lanka/COV53/2020	Original	First local patient in Sri Lanka Age range of 55–60 years	EPI_ISL_428671	2020-03-10	29 903	Human	Sputum	Centre for Dengue Research, University of Sri Jayewardenepura, Sri Lanka	GISAID (https://www.gisaid.org/, last access 28 April 2020; Supplementary material)
hCoV-19/Sri Lanka/COV38/2020	Original	Original age range of 30–33 years	EPI_ISL_428670	2020-03-16	29 903	Human	Sputum		
hCoV-19/Sri Lanka/COV91/2020	Original	Original age range of 35–40 years	EPI_ISL_428672	2020-03-19	29 903	Human	Sputum		
hCoV-19/Sri Lanka/COV486/2020	Original	Original age range of 45–50 years	EPI_ISL_428673	2020-03-31	29 903	Human	Oro-phalangeal swab		

### Selection of SARS-CoV-2 isolates

For further understanding of the molecular epidemiology of the COVID-19 outbreak in Sri Lanka, 46 isolates were selected from GenBank, National Center for Biotechnology Information (NCBI) using Basic Local Alignment Search Tool nucleotide (BLASTn) tool based on the highest identity and lowest expected value (E-Value) with Sri Lankan isolates. The sequence datasets of 46 selected SARS-Cov-2 complete genomes from different countries in Asia, Africa, Australia, Europe, North America and four Sri Lankan isolates retrieved from GISAID^5^ by 28 April 2020 were used for this analysis. The strain isolated from Wuhan in December 2019 with the NCBI accession number NC_045512.2 was used as the reference genome.

### Whole-genome sequence alignment and phylogenetic analysis

Sequence alignment was performed using Multiple Sequence Comparison by Log- Expectation (MUSCLE) software [7]. Following alignment, single nucleotide polymorphisms (SNPs) and amino acid variations analysis were conducted using Molecular Evolutionary Genetics Analysis version ten (MEGA X) [8], taking the first SARS-CoV-2 reference sequence (GenBank Accession number NC_045512) deposited December 2019 in GenBank from Wuhan, China. The evolutionary history was inferred using the neighbour-joining method with the maximum composite likelihood method and the Hasegawa–Kishino–Yano model (HKY) as the best fitting model [9] after 1000 bootstrap replication using MEGA X [8].

## Results

### Phylogenetic tree analysis

**Fig. 1.**
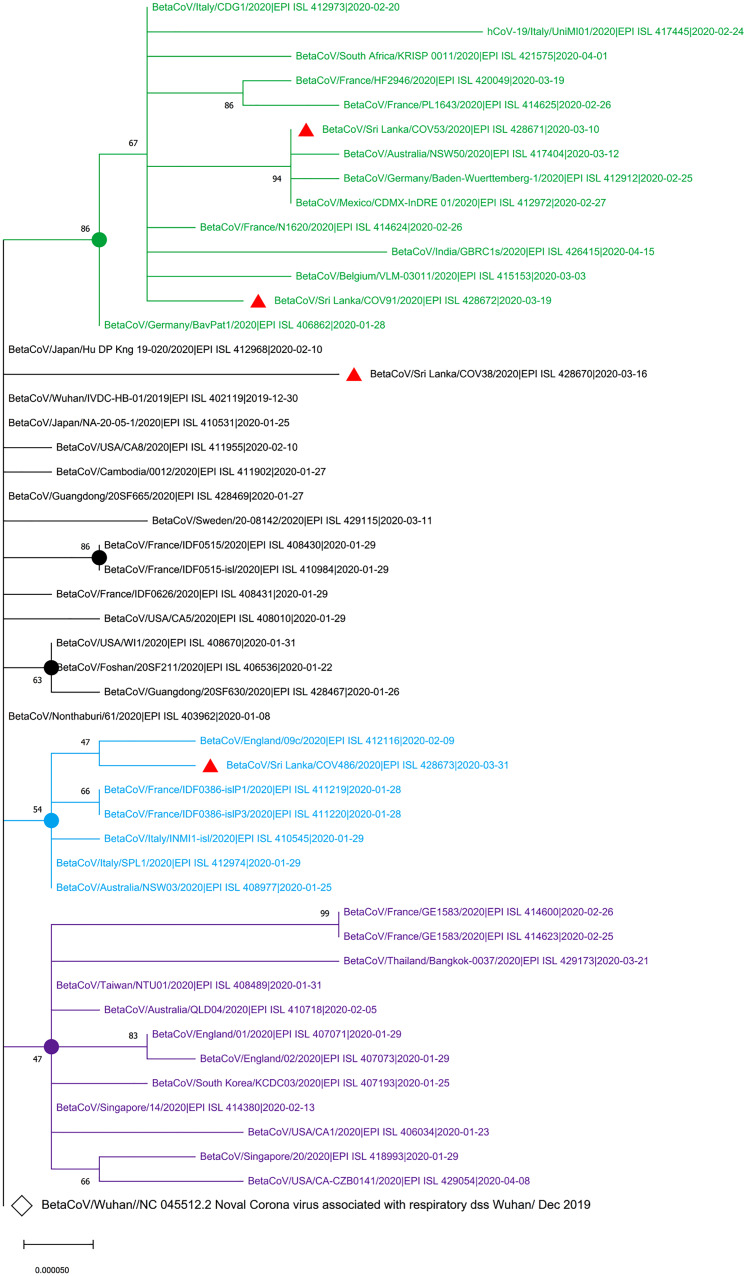
Phylogenetic analysis of four SARS-CoV-2 complete genome sequences of Sri Lanka retrieved in this study, with available selected complete genome sequences from different countries (*n* = 50 genome sequences). Strains names were written name followed by country of origin, GISAID accession number and sample collection date. GISAID: Global Initiative on Sharing All Influenza Data; HKY: Hasegawa, Kishino and Yano; MEGA X: Molecular Evolutionary Genetics Analysis across Computing Platforms; SARS-CoV-2: severe acute respiratory syndrome coronavirus. Sequence data obtained from GISAID: Global Initiative on Sharing All Influenza data. All Sri Lankan SARS CoV2 isolates are indicated in red triangles (
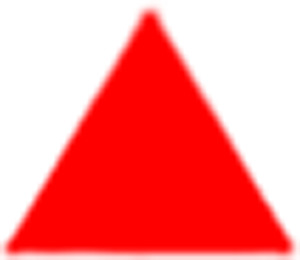
). The main clusters are highlighted in different colours. The Wuhan reference genome is in larger font (GenBank accession number: NC_045512.2). The filled circles represent the main supporting clusters and bootstrap support values are indicated at the level of the nodes. The tree was built using the best-fitting substitution model (HKY) through MEGA X software [7].

The maximum likelihood phylogenetic tree in Figure 1 shows that two of the SARS-CoV-2 isolates from Sri Lanka (GISAID accession IDs: EPI_ISL_428671 and EPI_ISL_428672) collected on 10 March 2020 and 19 March 2020, respectively, are clustered in the group with the isolates from Italy, Germany, France and Mexico that were collected before 10 March 2020. The EPI_ISL_428673 Sri Lankan isolates collected on 31 March 2020 was clustered with isolates obtained on 9 February 2020 from England while EPI-ISL_428670 Sri Lankan isolates collected on 16 March 2020 showed the highest evolutionary distance to the SARS-CoV-2 sequence originated in Wuhan, China (GenBank Accession number NC_405512).

### SNPs analysis

Fifteen SARS-CoV-2 genome sequences that are mainly clustered with the four Sri Lankan strains were compared with the Wuhan reference to observe the viral genome mutations and amino acid variations. The SNPs presented along with the whole genome are indicated in Table 2 (positions referred with respect to the reference sequence; GenBank accession number: NC_045512). The genome sequence of EPI_ISL_428671 from the first local patient has differed in six nt positions compared to the reference genome, while the rest of the three Sri Lankan sequences EPI_ISL_428670, EPI_ISL_428672 and EPI_ISL_428673 showed variations in six nt positions, five nt positions and four nt positions, respectively (Table 2). Both EPI_ISL_428671 and EPI_ISL_428672 strains, which clustered with the main group in the phylogenetic tree with European isolates, have shown three similar SNPs at the positions of bps3037, bps14408 and bps23403 (Table 2).

**Table 2.** Single nucleotide polymorphisms (SNPs)^a^ indicated in red colour constructed by comparison of four Sri Lankan whole-genome sequences of SARS-CoV-2 with selected SARS-CoV-2 sequences (*n* = 15 compared sequences)

	313	1059	1397	1762	2480	2558	3037	3467	4201	5986	6466	10 265	11 083	12 778	14 408	14 805	16 376	17 247	18 984	20 483	22 258	22 374	23 403	25 563	26 144	26 181	26 527	28 396	28 688	28 881	28 882	28 883	29 451	29 465

SARS-CoV-2 sequence ID (country from which the sequence originated)	ORF 1ab gene	ORF 1ab gene	ORF 1ab gene	ORF 1ab gene	ORF 1ab gene	ORF 1ab gene	ORF 1ab gene	ORF 1ab gene	ORF 1ab gene	ORF 1ab gene	ORF 1ab gene	ORF 1ab gene	ORF 1ab gene	ORF 1ab gene	ORF 1ab gene	ORF 1ab gene	ORF 1ab gene	ORF 1ab gene	ORF 1ab gene	ORF 1ab gene	Gene S	Gene S	Gene S	ORF3a gene	ORF3a gene	ORF3a gene	Gene M	Gene N	Gene N	Gene N	Gene N	Gene N	Gene N	Gene N
China/Wuhan/NC_405512	C	C	G	C	A	C	C	G	G	C	A	G	G	C	C	C	C	T	G	C	T	A	A	G	G	T	C	G	T	G	G	G	C	C
China/Wuhan/EPI_ISL_402119	C	C	G	C	A	C	C	G	G	C	A	G	G	C	C	C	C	T	G	C	T	A	A	G	G	T	C	G	T	G	G	G	C	C
Sri_Lanka/EPI_ISL_428673	C	C	G	T	A	C	C	G	G	C	A	G	G	C	C	T	C	C	G	C	T	A	A	G	T	T	C	G	T	G	G	G	C	C
Sri_Lanka/EPI_ISL_428672	C	C	G	C	A	C	T	G	T	C	A	G	G	C	T	C	C	T	G	C	T	A	G	G	G	T	T	G	T	G	G	G	C	C
Sri_Lanka/EPI_ISL_428671	C	C	G	C	A	C	T	G	G	C	A	G	G	C	T	C	C	T	G	C	T	A	G	G	G	T	C	G	T	A	A	C	C	C
Sri_Lanka/EPI_ISL_428670	C	C	A	C	A	C	C	G	G	T	A	G	T	C	C	C	C	T	C	T	T	A	A	G	G	G	C	G	C	G	G	G	C	T
England/EPI_ISL_412116	C	C	G	C	G	T	C	G	G	C	A	G	G	C	C	T	C	T	G	C	T	A	A	G	T	T	C	G	T	G	G	G	C	C
India/EPI_ISL_426415	C	C	G	C	A	C	T	T	G	C	G	G	G	C	T	C	C	T	G	C	T	G	G	G	G	T	C	A	T	G	G	G	T	C
Germany/EPI_ISL_406862	C	C	G	C	A	C	T	G	G	C	A	G	G	C	C	C	C	T	G	C	T	A	G	G	G	T	C	G	T	G	G	G	C	C
Italy/EPI_ISL_412973	C	C	G	C	A	C	T	G	G	C	A	G	G	C	T	C	C	T	G	C	T	A	G	G	G	T	C	G	T	G	G	G	C	C
France/EPI_ISL_420049	C	T	G	C	A	C	T	G	G	C	A	G	G	C	T	C	C	T	G	C	T	A	G	T	G	T	C	G	T	G	G	G	C	C
South Africa/EPI_ISL_421575	C	C	G	C	A	C	T	G	G	C	A	G	G	A	T	C	T	T	G	C	G	A	G	G	G	T	C	G	T	G	G	G	C	C
Mexico/EPI_ISL_412972	C	C	G	C	A	C	T	G	G	C	A	G	G	C	T	C	C	T	G	C	T	A	G	G	G	T	C	G	T	A	A	C	C	C
Germany/EPI_ISL_412912	C	C	G	C	A	C	T	G	G	C	A	A	G	C	T	C	C	T	G	C	T	A	G	G	G	T	C	G	T	A	A	C	C	C
Australia/EPI_ISL_417404	T	C	G	C	A	C	T	G	G	C	A	G	G	C	T	C	C	T	G	C	T	A	G	G	G	T	C	G	T	A	A	C	C	C

N: nucleocapsid protein; ORF: open reading frame; ORF1ab: ORF encoding polyprotein; S: surface glycoprotein; SARS-CoV-2: severe acute respiratory syndrome coronavirus; SNP: single nucleotide polymorphism; UTR: untranslated region.

SNPs are indicated in red colour.

a SNPs are shown according to nucleotide positions in the genome sequence and gene location.

### Amino acid variations

Table 3 indicates the respective changes in the amino acid positions of the derived proteins (positions referred with respect to the reference sequence; GenBank accession number: NC_045512). SNPs occurred only in the open reading frame (ORF) 1ab gene, S gene, ORF 3a gene, M gene and N gene of four Sri Lankan whole-genome strains have resulted in amino acid changes at the corresponding positions of the translated proteins, while the rest of SNPs in the genes did not result in any changes in amino acid sequence (Table 3).

**Table 3.** Amino acid variations^a^ construed by comparing translations of four Sri Lankan whole-genome sequences of SARS-CoV-2 with those of selected SARS-CoV-2 sequences (*n* = 15 compared sequences)

	265	378	739	765	1068	1312	3334	3606	4171	4847	5371	5661	6240	271	614	57	251	2	203	204	393	398

SARS-CoV-2 sequence ID (country from which the sequence originated)	ORF 1ab	ORF 1ab	ORF 1ab	ORF 1ab	ORF 1ab	ORF 1ab	ORF 1ab	ORF 1ab	ORF 1ab	ORF 1ab	ORF 1ab	ORF 1ab	ORF 1ab	S glycoprotein	S glycoprotein	ORF 3a	ORF 3a	M protein	N protein	N protein	N protein	N protein
China/Wuhan/NC_405512	T	V	I	P	G	M	G	L	Y	T	R	V	W	Q	D	Q	G	A	R	G	T	A
China/Wuhan/EPI_ISL_402119	T	V	I	P	G	M	G	L	Y	T	R	V	W	Q	D	Q	G	A	R	G	T	A
Sri_Lanka/EPI_ISL_428673	T	V	I	P	G	M	G	L	Y	I	R	A	W	Q	D	Q	V	A	R	G	T	A
Sri_Lanka/EPI_ISL_428672	T	V	I	P	G	I	G	L	Y	T	R	V	W	Q	G	Q	G	V	R	G	T	A
Sri_Lanka/EPI_ISL_428671	T	V	I	P	G	M	G	L	Y	T	R	V	W	Q	G	Q	G	A	K	R	T	A
Sri_Lanka/EPI_ISL_428670	T	I	I	P	G	M	G	F	Y	T	R	V	S	Q	D	Q	G	A	R	G	T	S
England/EPI_ISL_412116	T	V	V	S	G	M	G	?	Y	I	R	V	W	Q	D	Q	V	A	R	G	T	A
India/ EPI_ISL_426415	T	V	I	P	*	M	G	L	Y	T	R	V	W	R	G	Q	G	A	R	G	I	A
Germany/ EPI_ISL_406862	T	V	I	P	G	M	G	L	Y	T	R	V	W	Q	G	Q	G	A	R	G	T	A
Italy/ EPI_ISL_412973	T	V	I	P	G	M	G	L	Y	T	R	V	W	Q	G	Q	G	A	R	G	T	A
France/ EPI_ISL_420049	I	V	I	P	G	M	G	L	T	T	R	V	W	Q	G	H	G	A	R	G	T	A
South Africa/ EPI_ISL_421575	T	V	I	P	G	M	G	L	*	T	C	V	W	Q	G	Q	G	A	R	G	T	A
Mexico/ EPI_ISL_412972	T	V	I	P	G	M	G	L	Y	T	R	V	W	Q	G	Q	G	A	K	R	T	A
Germany/ EPI_ISL_412912	T	V	I	P	G	M	S	L	Y	T	R	V	W	Q	G	Q	G	A	K	R	T	A
Australia/ EPI_ISL_417404	T	V	I	P	G	M	G	L	Y	T	R	V	W	Q	G	Q	G	A	K	R	T	A

N: nucleocapsid protein; ORF: open reading frame; ORF1ab: ORF encoding polyprotein; S: surface glycoprotein; SARS-CoV-2: severe acute respiratory syndrome coronavirus; SNP: single nucleotide polymorphism; UTR: untranslated region.

*Stop codon.

? Possible sequencing error Amino acid variations are indicated in red colour.

a SNPs are shown according to nucleotide positions in the genome sequence and gene location. The amino acid positions refer to those in each respective protein sequence of the Wuhan reference (GenBank accession number: NC_405512), starting from the first methionine.

Except for the first Sri Lankan isolate collected on 10 March (EPI_ISL_428671), the other three Sri Lankan isolates presented a total of six mutations in the ORF 1ab protein with respect to the reference (Table 3). Mutations can be observed in the S protein at the same position AA614 (bps23403) in both Sri Lankan strains EPI_ISL_428671 and EPI_ISL_428672. A single mutation was observed in ORF 3a protein in strain EPI_ISL_428673 at the positions AA251 and bps26144. In the EPI_ISL_428670 strain, the amino acid sequence of N protein shows one mutation at the position AA398 at bps29465, while EPI_ISL_428671 strain had mutations at the positions AA203 (bps28882) and AA204 (bps28883) compared to the reference strain.

## Discussion

In this study, the virus strain of the first local patient (EPI_ISL_428671) collected on 10 March 2020, who was a tour guide and had direct contact with Italian tourists [4], is clustered together with isolates from Italy, Germany and Mexico. This evolutionary evidence revealed the first sequence of SARS-CoV-2 showed the highest genomic similarity to Italy, and European isolates confirming the history of exposure of the first patient who has been exposed to tourists who came from Italy. Even though the history and origin of the infection of the remaining isolates were not reported, the clustering of other isolates from Sri Lanka with isolates from the database has provided a clue about the possible source of the infection. Furthermore, genomic relatedness of the SARS-CoV-2 virus genome sequences of Sri Lankan isolates further confirmed the exposure history of the patients presented in the Epidemiology Unit, Ministry of Health, Sri Lanka [7]. More importantly, this study has indicated the importance of tracking the history of the infection to trace the contacts of the infected person, particularly for the asymptomatic patients in Sri Lanka. The mutations found in the virus identified in Sri Lanka, compared with the reference Wuhan strain and the recognise amino acid changes, should be further monitored to understand whether those changes affect the virulence of the virus or clinical manifestations of the disease. Though this study had limitations mainly due to the lack of epidemiological information on the available genome in the database and a limited number of sequence genome available in Sri Lanka at the time of analysis, the information obtained from this study might assist in understanding the evolutionary dynamics and local transmission of circulating SARS-CoV-2 in Sri Lanka.

## Conclusion

In this section, the results of this study indicated that the SARS-CoV-2 sequences from Sri Lanka have the highest genomic similarity to isolates from Italy, Germany and England. This study was conducted as a preliminary study in Sri Lanka; further studies are necessary to be performed to increase our knowledge regarding SARS-CoV-2 isolates. Since the mutational variants can alter the presentation of COVID-19 infection, the robust molecular epidemiological tools indicated in this study can be used to trace possible exposure, epidemiological analysis and develop an effective treatment, including vaccines.

## Data Availability

Data and material are provided in the supplementary material and available at www.gisaid.org.

## Supplementary material

For supplementary material accompanying this paper visit http://dx.doi.org/10.1017/S0950268821000583. click here to view supplementary material
